# Illinois State Board of Health—Abstract of Report of Quarterly Meeting

**Published:** 1888-09

**Authors:** 


### EXTRACTS AND ABSTRACTS.

### ILLINOIS STATE BOARD OF HEALTH.

AN ABSTRACT OF PROCEEDINGS AT THE QUARTERLY MEETING IN CHICAGO, JUNE 28–29, 1888.

The minutes of the last regular meeting, April, 1888, were read and approved.

The reading of the Secretary's quarterly report was deferred until the following day.

In the matter of certain applications for certificates under the Medical Practice Act, the time was extended for parties to make reply to charges filed against them.

Representatives of two medical colleges, against which charges had been filed, appeared before the Board and submitted arguments to show that their institutions should be considered in "good standing" by the Board. In both cases the matter was taken under advisement. Consideration of cases under the Medical Practice Act occupied the attention of the Board during the remainder of the first day's session.

Pursuant to adjournment, the Board resumed its session at 10 o'clock a. m. June 29.

Under the regular order of business the Secretary presented his quarterly report, in which he said: Although there have been nine importations of small-pox into the State during the year, and the disease is prevalent in many surrounding localities, and especially among the negroes along the Mississippi and Ohio Rivers, there has been no spread of the contagion in Illinois, and the outbreaks thus far have been promptly suppressed with only one exception. The two cases in Bond County, at Greenville and at Reno—existing at the date of the last report in April—caused no others in the neighborhood, and up to the latter part of May the State was free from the disease. In May three cases were reported at Metropolis, in Massac County, and in June one at Kaskaskia.

In the early part of the quarter there were localized outbreaks of scarlet fever at Nokomis, Earlville, Nashville, Biggsville, Foreston, and Kinderhook; of German measles at Plainfield and Kinderhook; of diphtheria at Earlville, and of typhoid fever at Mt. Pulaski and Springfield. This latter occurrence will be made the subject of a detailed report to the Board at a later date, when the investigations now being made—and including chemical and biological examinations of the water supply—shall have been completed. The outbreak occurred among the students of Concordia College, some of whom had nursed a little negro waif sick with the disease. It resulted in forty-five cases and nine deaths. The session was interrupted, the classes broken up, and the institution will not be again opened until the premises are put in good sanitary condition.

GENERAL SANITARY WORK.

Aside from the foregoing, the general health of the State has been very satisfactory. A good amount of sanitary work has been accomplished, a number of cities and towns having completed their re-inspections, and others having begun this work for the first time. Galesburg, Cairo, and Sparta are to be especially commended for the thoroughness of their work in this connection.

The circular of the Board addressed to railway managers in April last has met with a very gratifying reception. Many roads have entirely reformed the sanitary conditions with respect to the accommodations for passengers at stations; vaults have been emptied, disinfected, and the contents disposed of so as to avoid future nuisance; and arrangements are now secured for keeping these appurtenances in a cleanly and comfortable condition. Much attention has also been given to the purity of the drinking water furnished at the stations and on the trains.

Work on the investigation of the water supplies of the State has been impeded to some extent by the high stage of water in many of the streams, and lately by the excessive rainfall over large areas. Considerable progress, however, has been made. Time-tables for the collection of water supplies at various points—twenty-three in number—between Chicago and Alton have been constructed, so as to make it practicable to follow the changes, by pollution and purification, in a given body of water from the time it leaves Lake Michigan until it flows past the city of Alton. Reports of the chemical and biological examinations are received in the Secretary's office.

Except with the interruptions noted, samples of water are now collected and examined once a week at Chicago (lake water), Bridgeport, Lockport, Joliet (above and below city), Morris, La Salle, Henry, Peoria, Pekin, Copperas Creek Dam, Havana, Beardstown, Pearl, and Grafton, on the main stream; and from the Kankakee, Du Page, Fox, Big Vermilion and Little Vermilion, Sangamon and Spoon tributaries. Samples will also be taken from Alton and East St. Louis (Mississippi river water), and from other points as found necessary in the course of the work, which will continue six months or more.

In each sample the following determinations are made: Total solids, suspended matter, nitrates and nitrites, chlorine, hardness, free ammonia, albuminoid ammonia, oxygen consumption, and color and odor. By continuing the work through so long a period, and by making the analyses as fully as outlined, it is hoped that the effect of accidental variations will be practically eliminated. The amount of flow from some of the tributaries is pretty well known, and before the end of the summer it will be known for all, so that we will then be in possession of data complete enough to enable us to give a fair answer to the question, What is the rate of oxidation in the Illinois river in a flow of four hundred miles, with sources and amount of contamination, and amount of dilution from tributaries, practically known.

The results obtained thus far in this investigation show the importance of a study of the tributaries. The waters of some of these appear to contain a great deal of decaying vegetable matter. The character of these waters will be thoroughly studied, as an understanding of them is of the highest importance to the sanitary welfare of the communities supplied from such sources. The public water supplies of some of the cities and towns are also being investigated.

The investigation of the underground water supplies is making satisfactory progress—records from some sixty different localities, in all parts of the State, having been already secured.

CONFERENCE OF STATE BOARDS OF HEALTH.

In conformity with the instructions of the Board, the Secretary attended the session of the National Conference of State Boards of Health in Cincinnati, May 4 last. The proceedings of this meeting have been published, and it is only necessary in this connection to refer to so much as is of especial interest to Illinois. A committee was established to visit or correspond with the State, Provincial and other authorities having charge of the sea-board quarantines against dangerous infectious diseases, for the purpose of learning the methods in use, and the amount of co-operation such authorities can and will give for the protection of the people of this continent against said diseases. The committee is also authorized to act for the conference for this purpose, and is instructed to report the results of its investigation to the conference and to the State Boards of Health, and to arrange for proper co-operation should any foreign epidemic disease threaten to invade or actually get a foothold on this continent.

The members of this committee are: Dr. J. H. Rauch, Illinois, chairman; Dr. H. B. Baker, Michigan; Dr. C. N. Hewitt, Minnesota; Dr. J. N. McCormack, Kentucky; Dr. T. G. Simons, South Carolina; Dr. James Simpson, California; Dr. John D. Jones, Ohio; Dr. P. H. Bryce, Province of Ontario, Canada, and Dr. Benjamine Lee, of Pennsylvania. Sub-committees were subsequently appointed to visit personally, where necessary, all the quarantine stations on the Atlantic and Pacific coasts, from the St. Lawrence to the Rio Grande, and from Oregon to Lower California; these sub-committees to secure the co-operation of the States should cholera or any other epidemic disease obtain a foothold.

The chairman of the general committee is a member *ex officio* of each of these sub-committees, and was requested by the members to formulate instructions as to the lines on which the investigations of the various quarantine establishments should be conducted. This has already been done, and in order to cover the ground more completely Drs. F. A. Gerrish, of Portland, Maine; C. A. Lindsley, of New Haven, Connecticut; H. S. Orme, of Los Angeles, California, and J. N. Evans, of South Carolina, have been added to the committee.

NATIONAL QUARANTINE.

Regulations approved by the President of the United States, and promulgated by the Secretary of State to United States consular officers, have been re-published for the information of United States quarantine, State and municipal health officers. These are based upon Section 2 of the Act of 1878, which provides that whenever any infectious or contagious disease shall appear in any foreign port or country, and whenever any vessel shall leave any infected foreign port, or, having on board goods or passengers coming from any place or district infected with cholera or yellow fever, shall leave any foreign port bound for any port in the United States, the consular officer or other representative of the United States at or nearest such foreign port shall immediately give information thereof through the Department of State, and shall report the name, the date of departure, and the port of destination in the United States; the object being to secure timely advice of the outbreaks of cholera and yellow fever, and of the probable transportation of the poisons of these preventable diseases in vessels bound for this country.

The Marine Hospital Service, through the Treasury Department, has also secured instructions to the Revenue Marine Service to co-operate in the prevention of the introduction of any foreign pestilence as far as practicable.

The maritime quarantine service is better administered at this time than ever before.

Thus far, although Asiatic cholera still exists in South America, there has been no intimation of its reappearance in any of the districts of Europe which were infected last year, nor of its development in any of the countries which were then threatened.*

CONFERENCE OF LOCAL HEALTH AUTHORITIES.

The conference of health authorities of cities, towns and villages of the State, for the purpose of promoting the work of local sanitation, which was authorized at the April quarterly meeting, was held at the State capitol on May 17–18.

Professor O. C. De Wolf made an address upon the "Disposal of Garbage"; Mr. Genung upon the "Plumbing and Drainage of Houses"; Professors Long and Curtis upon "Water-Supply Pollution"; Dr. Buckley read a paper on the "Disposal of Nightsoil"; and Dr. Cohen upon the "Methods of Prevention and Suppression of Epidemics of the Contagious and Infectious Diseases."

The Secretary reported on the "Registration of Vital Statistics," and the attempts of the State Board to secure better legislation on the subject. It was shown that too much is asked of the physician under the present system.

The necessity for a uniform health organization for cities and towns throughout the State led to the adoption of the following resolution:

"Whereas, Uniformity in the organization of the various boards of health of this State, together with having their powers and duties clearly defined by law, with the full and clearly defined means of enforcing the order of said boards, is a matter of such importance as to demand the attention of our law-makers; therefore be it

"*Resolved*, That a committee of five be appointed to prepare a bill for an act to create boards of health in the several cities and villages of the State, defining their powers and duties and fixing their compensation, and cause the same to be presented to the next General Assembly of this State, and urge its passage either as an amendment to the present act for the incorporation of cities and villages, or as an independent law."

MEDICAL EDUCATION AND STATISTICS.

In justification of the general acceptance, both in this country and abroad, of the conclusions in the reports of the Illinois State Board of Health on the subject of Medical Education in the United States and Canada—and with especial reference to the accuracy of the statistics contained in those reports—the following passages in the address of the President of the American Medical Association, delivered at the Thirty-ninth Annual Meeting, on the 8th of May last, are noted.

"That the professions of law and medicine are overcrowded in this country, no man of common observation will deny. The ratio of professional men in the

* Since the date of the meeting, two deaths from cholera are reported at Messina, Italy.

United States to the population exceeds that of any other country in the civilized world, so that any legitimate means of checking this evil which can be devised and carried into practical effect must be hailed by the medical world, as well as the general public, as an inestimable boon. The statistics recently published by the Illinois State Board of Health tending to show the annual number of graduates in medicine within the last three years, and the conclusions based upon those statistics by the medical journals of the country, I have reason to believe, are not entirely accurate. In support of this opinion and the views expressed in the foregoing part of this address, I shall take the liberty of directing your attention to a résumé of statistics bearing upon this subject furnished me through the kindness and courtesy of Hon. N. H. R. Dawson, United States Commissioner of Education.

"These will, no doubt, exhibit in a more forcible and impressive manner to your minds the special subject under consideration than any individual argument, however logical or conclusive, that could be presented, particularly when it is remembered that they are prepared under the immediate authority and direction of the United States Government, having at its command far greater resources than any one State or county could employ."

So far as it is possible to discover in the extract from the "Résumé of statistics"—for it is obviously only an extract which is furnished in the address—so far as appears from this extract the figures of the Commissioner of Education relate to a single year, and not to "the annual number of graduates in medicine within the last three years."

This appears to be the college year 1885–86, during which the Commissioner found "89 regular, 10 eclectic, 13 homœopathic, and 2 physio-medical schools" in existence—in the sense of holding sessions and graduating students.

The Board's report, however, includes all colleges in existence in 1885–86, while the Commissioner's figures are intended to include only those which had classes of graduates.

Each succeeding year's catalogues and announcements are carefully examined; where the names of matriculates are published these are compared name by name, and all duplicates are deducted from the total. It may be explained that the desire to show a large attendance, and also to reduce the apparent proportion of graduates to matriculates, leads a certain class of schools to this duplication of names.

The "Forty-fourth Annual Catalogue and Announcement" of one such school, in which there are 46 duplicates in a claimed total of 216 matriculates in attendance upon the sessions of 1886-87, was received by the Board. The number of graduates was 63, and the claimed proportion of graduates to matriculates is 29 per cent. It should be 37 per cent. The year before there were 56 duplicates detected out of 217 names; total graduates 58; claimed proportion 26.7 per cent.; actual proportion 36 per cent. This year the names of matriculates are not published; but, as in all similar cases, the college will be requested not only to furnish the names in writing for the information of the Board, and to enable it to determine the recognition of its diplomas, but the affidavit will be exacted which the Board now requires of all schools concerning which there is a question as to good faith in conforming to the standard of minimum requirements, or of enforcing their own published regulations, or of furnishing the instruction and facilities advertised.

It must be obvious that statistics compiled, and data accumulated and treated in the manner herein indicated,* have a measure of value and accuracy not attainable by any agency restricted to the purely vol-

* Before the publication of each issue of the report advance-sheets, "proofs," or copies of the immediately preceding report are sent to the proper officers of every institution concerned, with a request for examination, for the correction of any inaccuracies, and for the addition of any new matter pertinent to the subject.

untary information contributed from interested sources and over which the agency may exercise no control. The importance of the recognition of a medical college as in "good standing" in Illinois, under the Medical Practice Act of the State, is felt throughout the entire country, as there is not a medical college that does not desire the recognition of its diplomas by this Board. It is this which has made it practicable to issue a series of reports on medical education and medical institutions, the trustworthiness and utility of which have not only never heretofore been questioned, but which have been widely recognized both in this country and abroad as exercising a marked influence in raising the standard of medical instruction and of the qualifications necessary for the practice of medicine, and thereby improving the status of the medical profession, with consequent benefit to the public welfare.

MEDICAL PRACTICE ACT.

During the quarter fines of $100 each have been imposed upon five unqualified men who violated the law. Other suits are pending. It is now one year since the new law went into effect, and nearly all who were required to take out certificates have done so. The effect of this law, especially with regard to itinerant showmen and mountebanks who sold medicines, has been marked and gratifying. The Board refused to grant licenses to this class, believing their business to be prejudicial to the interests of the people, and in so doing refused $11,400 in license fees. During the month of June, 1887, there were twenty-two itinerant companies in the State whose sales daily amounted to about $2,000. It is safe to assume that this law has saved the people of the State not less than $250,000 during the past year.

In the matter of the recognition of the diplomas of a medical college, taken under advisement at Thursday's session, consideration thereof was resumed, and on motion it was decided not to issue certificates to three graduates of said school whose applications were pending, for the reason that the school had not in their cases complied with its own printed requirements or the requirements of this Board with respect to preliminary education.

